# The Dual-Specificity Phosphatase 2 (DUSP2) Does Not Regulate Obesity-Associated Inflammation or Insulin Resistance in Mice

**DOI:** 10.1371/journal.pone.0111524

**Published:** 2014-11-06

**Authors:** Graeme I. Lancaster, Michael J. Kraakman, Helene L. Kammoun, Katherine G. Langley, Emma Estevez, Ashish Banerjee, Raelene J. Grumont, Mark A. Febbraio, Steve Gerondakis

**Affiliations:** 1 Cellular and Molecular Metabolism Laboratory, Baker IDI Heart and Diabetes Institute, Melbourne, Victoria, Australia; 2 The Australian Centre for Blood Diseases and Department of Clinical Hematology, Monash University Central Clinical School, Melbourne, Victoria, Australia; 3 Centre for Cancer Research, MIMR-PHI Institute of Medical Research, Monash University, Victoria, Australia; National Cancer Institute, United States of America

## Abstract

Alterations in the immune cell profile and the induction of inflammation within adipose tissue are a hallmark of obesity in mice and humans. Dual-specificity phosphatase 2 (DUSP2) is widely expressed within the immune system and plays a key role promoting immune and inflammatory responses dependent on mitogen-activated protein kinase (MAPK) activity. We hypothesised that the absence of DUSP2 would protect mice against obesity-associated inflammation and insulin resistance. Accordingly, male and female littermate mice that are either wild-type (*wt*) or homozygous for a germ-line null mutation of the *dusp2* gene (*dusp2^−/−^*) were fed either a standard chow diet (SCD) or high fat diet (HFD) for 12 weeks prior to metabolic phenotyping. Compared with mice fed the SCD, all mice consuming the HFD became obese, developed glucose intolerance and insulin resistance, and displayed increased macrophage recruitment and markers of inflammation in epididymal white adipose tissue. The absence of DUSP2, however, had no effect on the development of obesity or adipose tissue inflammation. Whole body insulin sensitivity in male mice was unaffected by an absence of DUSP2 in response to either the SCD or HFD; however, HFD-induced insulin resistance was slightly, but significantly, reduced in female *dusp2^−/−^* mice. In conclusion, DUSP2 plays no role in regulating obesity-associated inflammation and only a minor role in controlling insulin sensitivity following HFD in female, but not male, mice. These data indicate that rather than DUSP2 being a pan regulator of MAPK dependent immune cell mediated inflammation, it appears to differentially regulate inflammatory responses that have a MAPK component.

## Introduction

Research over the last two decades has identified that chronic, low-grade inflammation plays a critical role in initiating insulin resistance, a key step in the development of type 2 diabetes [1]. Underpinning obesity-associated WAT inflammation is a marked shift in the immune cell profile of the WAT during the transition from a lean to obese state [2]. A loss of anti-inflammatory immune cells, concomitant with the accumulation of pro-inflammatory immune cells, initiates a state of local inflammation within the WAT that contributes to insulin resistance [2]. Studies in which particular immune cells or pro-inflammatory molecules have been genetically or pharmacologically targeted underscore the important role specific immune cells, and inflammation more generally, play in systemic insulin resistance [3]–[8].

Amongst the different classes of molecules shown to influence metabolism are protein tyrosine phosphatases [9]. The protein tyrosine phosphatase (PTP) superfamily is divided into classical PTPs and dual specificity phosphatases (DUSPs), the products of which catalyze the hydrolysis of phospho-tyrosine and phospho-threonine substrates [10]. Classical PTPs such as PTP1B and T-cell PTP have been identified as critical regulators of insulin and leptin signalling, serving important roles in the development of obesity and insulin resistance [11]–[14]. Of the many DUSPs described to date are the class I sub-family of DUSP proteins. The class I DUSPs specifically recognize and de-phosphorylate the conserved TxY motif present in the activation loop of the 4 major classes of mitogen-activated protein kinases (MAPK); ERK (1 and 2), ERK5, p38 (α, β, γ and δ) plus JNK (1, 2 and 3), and, accordingly, are also referred to as MAPK phosphatases (MKPs) [15]. Based on sequence homology, substrate specificity and subcellular localization, these MKPs can be further sub-divided into three groups. The first includes DUSP1/MKP-1, DUSP2 (PAC1), DUSP4/MKP-2 and DUSP5, all of which serve as mitogen- and stress-inducible nuclear MKPs. The second group includes DUSP6/MKP-3, DUSP7/MKP-X and DUSP9/MKP-4, which are ERK-specific MKPs localized exclusively in the cytoplasm. The final group comprising DUSP8 (M3/6), DUSP10/MKP-5 and DUSP16/MKP-7 are JNK/p38-specific phosphatases found in both the cytoplasm and nucleus [10].

Consistent with the numerous roles MAPKs serve regulating diverse processes in immune cells that encounter growth factors, pathogens and inflammatory cytokines or undergo stress [10], DUSPs provide a critical level of spatiotemporal control over these responses. For example, in the absence of either DUSP1 [16] or DUSP10 [17], lipopolysaccharide (LPS) treated macrophages display increased JNK activation and elevated pro-inflammatory cytokine production, whereas LPS activated macrophages isolated from DUSP2-deficient (*dusp2^−/−^*) mice exhibit reduced pro-inflammatory cytokine production [18]. The roles DUSPs play controlling MAPK-dependent innate immune and inflammatory responses has prompted an examination of their individual functions in the context of obesity and insulin resistance. Specifically, over-expression of DUSP9 decreases ERK and JNK phosphorylation and improves glucose tolerance in genetically obese ob/ob mice [19]. DUSP1-deficient mice by contrast do not have improved glucose tolerance, despite being protected from HFD-induced obesity [20].

However, the regulatory roles other DUSPs play in inflammation-induced obesity and insulin resistance remains unclear. One candidate of interest is DUSP2. First cloned as an immediate early gene in human T lymphocytes [21], DUSP2 belongs to the sub-family of nuclear specific DUSPs that includes DUSP1, 4 and 5 [10]. DUSP2 is primarily expressed within the immune system [21], [22] and is further up-regulated in diverse immune cells following activation [18], [22]. Importantly, a lack of DUSP2 in immune cells suppresses the production of a variety of pro-inflammatory mediators *in vitro* [18] and reduces inflammation and disease pathology in a murine model of inflammatory arthritis [18].

Based on the key role of immune cells and inflammation in obesity-associated insulin resistance, the roles ascribed to certain DUSPs in the regulation of obesity and insulin resistance, high level DUSP2 expression in various activated immune cells, plus reduced inflammation *in vitro* and *in vivo* in DUSP2-deficient cells, we have examined the role of DUSP2 in the context of obesity-associated inflammation and insulin resistance. We hypothesized that a loss of DUSP2 function would reduce obesity-associated inflammation and, consequently, would improve insulin sensitivity in mice fed a HFD.

## Materials and Methods

Generation of the *dusp2^−/−^* mice were described previously [18] and this strain has been backcrossed to the C57Bl6J background for>10 generations. Wild-type (*wt*) and the DUSP2 deficient (*dusp2^−/−^*) male and female littermates used in this study were generated by mating mice heterozygous for a *dusp2* null allele. Mice were housed at 22±1°C and maintained on a 12/12 h light/dark cycle, with free access to drinking water and food. Mice were fed either a SCD containing 5% of energy from fat or a HFD containing 43% of energy from fat (both diets were supplied by Specialty Feeds, Glen Forrest, WA, Australia). All procedures were approved by the AMREP Animal Ethics Committee and were in accordance with the National Health and Medical Research Council of Australia guidelines.

Bone marrow-derived macrophages (BMDM) were generated from the hind limb bones of *dusp2^−/−^* and *wt* C57Bl6J mice. After 7 days of culture in RPMI media containing 15% fetal bovine serum (FBS) and 20% L-cell conditioned media, BMDM were changed overnight to RPMI media containing 5% FBS and 2% bovine serum albumin (BSA) prior to experimental treatment. Palmitate (P0500; Sigma Aldrich) was dissolved in 100% ethanol at a stock concentration of 100 mM and was then conjugated to bovine serum albumin (BSA) (2% weight per volume; A6003; Sigma Aldrich) in RPMI media containing 5% FBS to create a final concentrations of 1 mM. The vehicle contained BSA and ethanol, but no palmitate. Media was collected after 8 h of treatment with either palmitate or vehicle and the concentrations of the pro-inflammatory cytokines IL-1β and TNF were then determined by enzyme-linked immunosorbent assay (R&D Systems, Minneapolis, USA). For the analysis of intracellular signaling molecules, cells were lysed after 4 h of treatment with palmitate or vehicle, protein concentrations determined and samples examined by Western blotting as described below.

Mice were administered 2 g of glucose per kg of lean mass by oral gavage following a 5 h fast to assess glucose tolerance. Blood was obtained prior to and at the indicated intervals for the proceeding 2 h from the tip of the tail. Blood glucose concentrations were determined using a glucometer (Accu-Check, Roche, NSW, Australia). Fat and lean mass were determined using an EchoMRI 4-in-1 (Echo Medical Systems, Houston, TX, USA). Plasma insulin concentrations were determined using a mouse ultrasensitive plasma insulin ELISA (ALPCO, Salem, NH, USA).

For quantitative RT-PCR, RNA was extracted from epididymal WAT using Trizol (Invitrogen, Carlsbad, CA, USA) and total RNA measured using the ND-1000 NanoDrop Spectrophotometer (Thermo Scientific, Waltham, MA, USA). RNA samples were treated with DNase I (Invitrogen) and cDNA generated using the Tetro cDNA synthesis kit (Bioline). Gene expression studies were performed by RT-PCR using TaqMan primers and probes for genes of interest and 18S rRNA (Applied Biosystems, Foster City, CA, USA). For cytokine and DUSP2 gene expression, 40 ng of cDNA was used, whereas *f4/80* and *cd11c* expression was determined using 20 ng of cDNA. The comparative C_T_ method was used to quantify results from RT-PCR.

Genotyping of tail or ear clip DNA samples was performed using the following primers. WT: Forward –5′-ATTTGCTCTCCCTTCTTCGA-3′ and Reverse 5′-TGACACACACACGTCACTTCCT-3′. DUSP2 KO: Forward 5′-ATTTGCTCTCCCTTCTTCGA-3′ and Reverse 5′-GCCTGCTCTTTACTGAAGGC-3′. The following cycling conditions were used: 1 cycle of 3 min at 95°C, 60 s at 57°C, 45 s at 72°C, followed by 39 cycles of 60 s at 95°C, 60 s at 57°C, 45 s at 72°C. Products were resolved on a 2% agarose gel containing ethidium bromide.

For Western blotting, epididymal WAT was removed from mice under anesthesia, snap frozen in liquid N_2_ and stored at −80°C. WAT was lysed and protein concentrations determined using the BCA method (Thermo Scientific). Samples (20 ug total protein per sample) were solubilized in Laemmli's buffer and heated at 95°C for 5 min, then western blotting performed as described previously [23]. Antibodies against phosphorylated (4671) and total (9258) forms of JNK, phosphorylated (9211) and total (9212) forms of p38, and total ERK1/2 (9102) were from Cell Signalling Technology Inc (Danvers, MA, USA). The antibody against phosphorylated ERK1/2 (sc-7383) was from Santa-Cruz Biotechnology, Inc.

Statistical analysis was performed using SigmaStat version 3.5. Statistical significance was determined using a Student's t-test or 2 way ANOVA analysis, where appropriate. Data are presented as the mean ± the standard deviation. A p-value of less than 0.05 was considered statistically significant; although we have indicated where the p-values were lower than 0.05. Quantification of western blots was done using Quantity One (Bio-Rad Laboratories).

## Results

**Figure 1:**
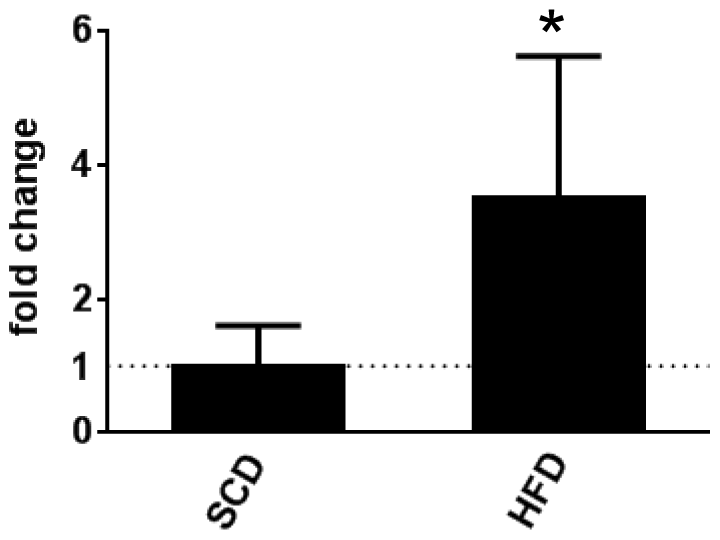
HFD increases *dusp2* mRNA expression. *Dusp2* mRNA expression in the WAT of *wt* C57Bl6J mice fed either SCD or HFD for 12 weeks. * p<0.05. Data are mean ± SD. N = 5 for SCD and 8 for HFD.

DUSP2 expression is markedly increased in many immune cell types following activation [18], [22]. Given activated immune cells accumulate in the adipose tissue of obese mice, we first determined whether *dusp2* gene expression was increased in the epididymal WAT of obese mice. Consistent with the links between immune cell dependent inflammation and obesity, *dusp2* mRNA expression was significantly higher in WAT from *wt* mice fed a HFD compared with *wt* mice fed a SCD (Figure 1).

**Figure 2:**
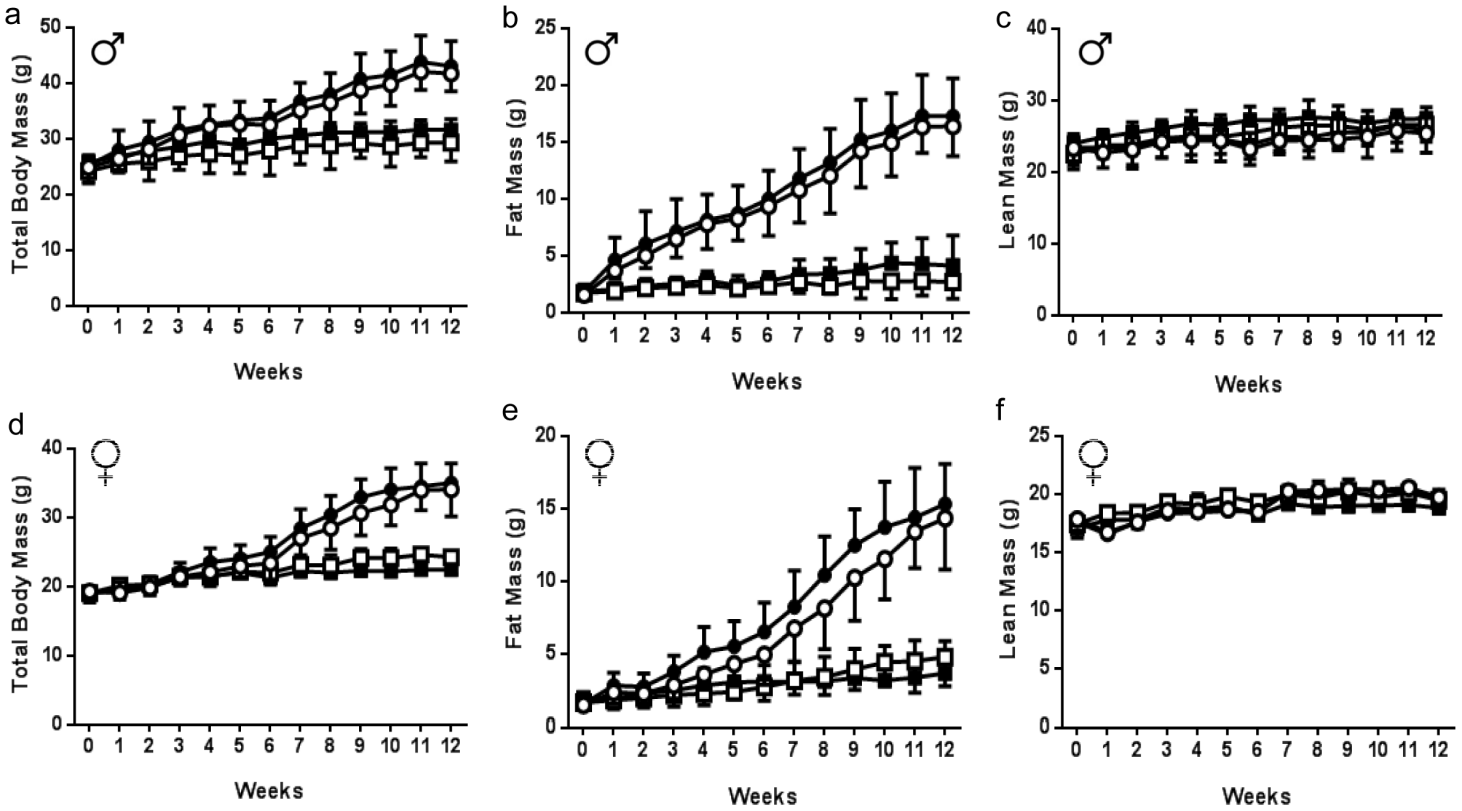
The development of obesity in *wt* and *dusp2^−/−^* male and female mice. (**a** and **d**) Total body mass; (**b** and **e**) fat mass; and (**c** and **f**) lean mass in *wt* and *dusp2^−/−^* male and female mice fed either a SCD or HFD for 12 weeks. Data are mean ± SD. White squares are *wt* mice fed SCD; Black squares are *dusp2^−/−^* mice fed SCD; White circles are *wt* mice fed HFD; Black circles are *dusp2^−/−^* mice fed HFD. For male mice, Ns are 5, 8, 8 and 8, for *wt* SCD, *dusp2^−/−^* SCD, *wt* HFD and *dusp2^−/−^* HFD, respectively. For female mice Ns are 9, 8, 7 and 7 for *wt* SCD, *dusp2^−/−^* SCD, *wt* HFD and *dusp2^−/−^* HFD, respectively.

To investigate the role of DUSP2 in the context of obesity-associated inflammation and insulin resistance, we exploited a mutant strain of mice that lack DUSP2 [18]. Male and female *wt* and *dusp2^−/−^* litter mates were fed either a SCD or HFD for 12 weeks commencing at 8 weeks of age. DUSP2-deficient mice have no known lymphoid or myeloid developmental abnormalities and have normal longevity free from any spontaneous immunopathology [18]. Both male (Figure 2a–c) and female (Figure 2 d–f) *wt* and *dusp2^−/−^* mice responded as expected to the HFD. The HFD resulted in an increase in total body mass (Figure 2a and d), an effect entirely attributable to an increase in fat mass (Figure 2b and e). While male mice appeared to gain fat mass more rapidly than female mice fed a HFD, at the end of the dietary period both male and female mice, irrespective of genotype, had gained similar amounts of fat mass (Figure 2b and e).

**Figure 3:**
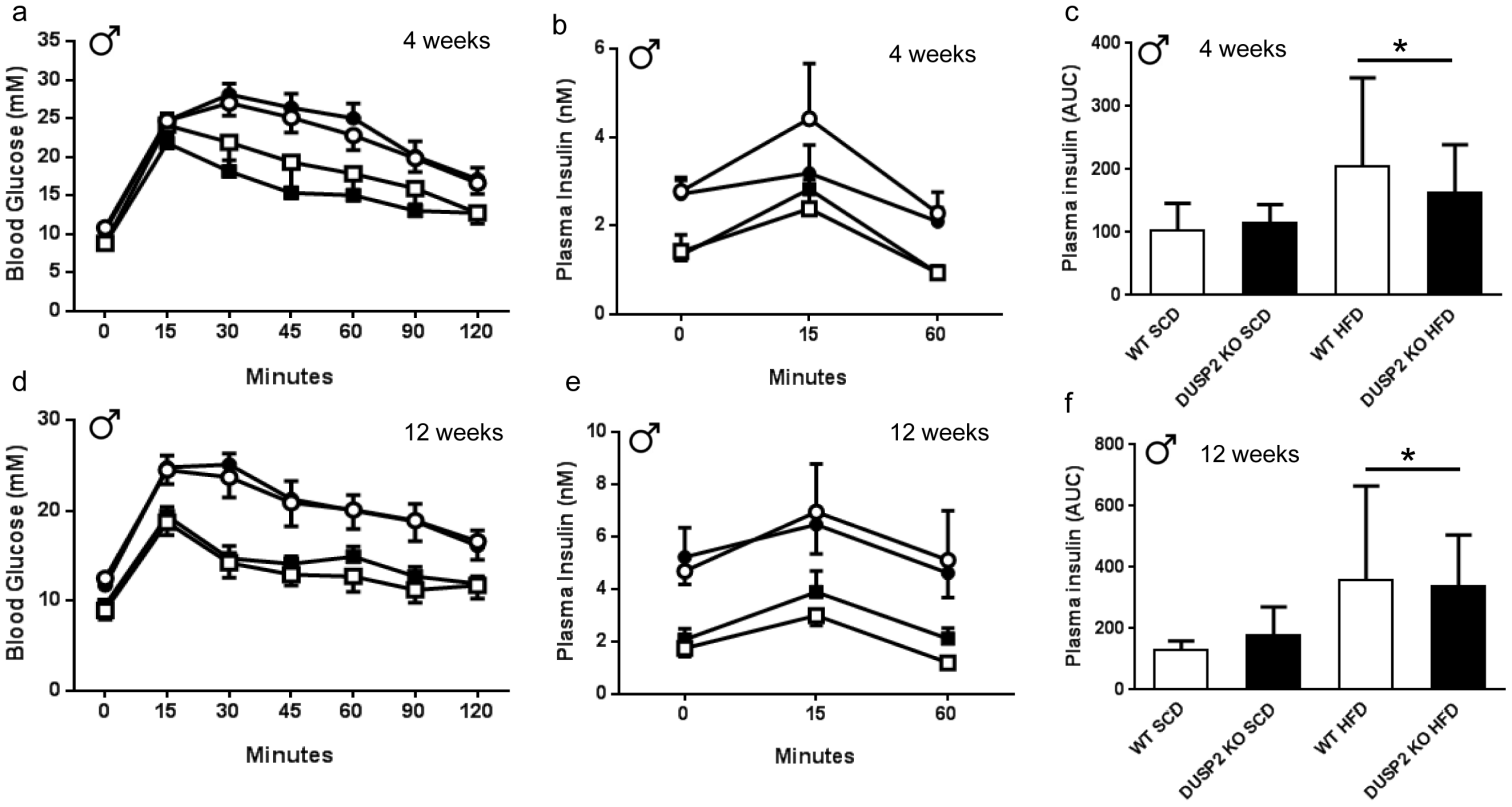
Glucose tolerance and insulin sensitivity in *wt* and *dusp2^−/−^* male mice after 4 and 12 weeks of diet. (**a** and **d**) oral glucose tolerance; (**b** and **e**) plasma insulin during the OGTT; and (**c** and **f**) plasma insulin area under the curve (AUC). * p<0.05; Main Effect for HFD vs SCD. Data are mean ± SD. White squares are *wt* mice fed SCD; Black squares are *dusp2^−/−^* mice fed SCD; White circles are *wt* mice fed HFD; Black circles are *dusp2^−/−^* mice fed HFD. Ns are 5, 8, 8 and 8, for *wt* SCD, *dusp2^−/−^* SCD, *wt* HFD and *dusp2^−/−^* HFD, respectively.

**Figure 4:**
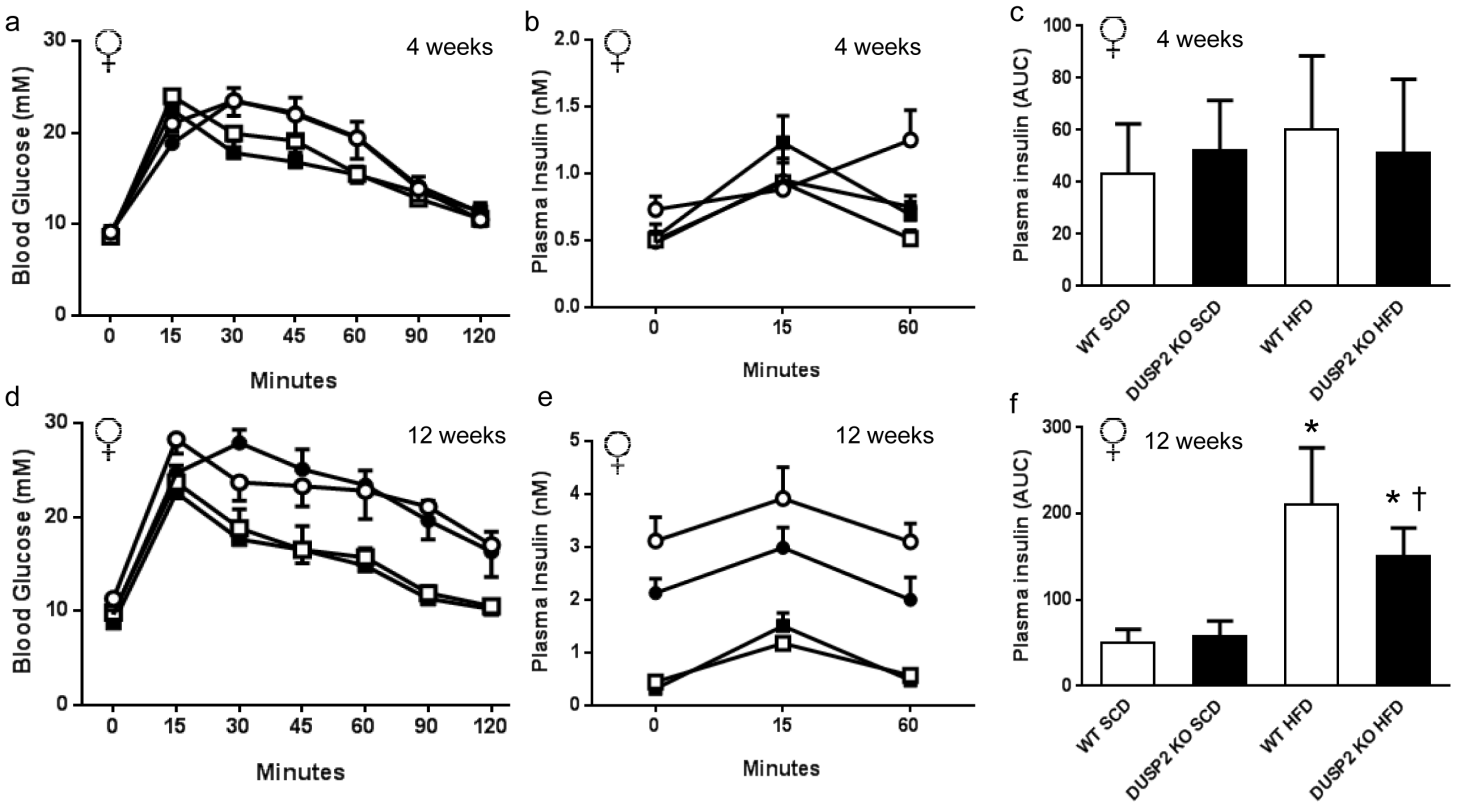
Glucose tolerance and insulin sensitivity in *wt* and *dusp2^−/−^* female mice. (**a** and **d**) oral glucose tolerance; (**b** and **e**) plasma insulin during the OGTT; and (**c** and **f**) plasma insulin area under the curve (AUC). *p<0.05; Interaction Effect *wt* SCD vs. *wt* HFD and *dusp2^−/−^* SCD vs. *dusp2^−/−^* HFD. † p<0.05 Interaction Effect *wt* HFD vs. *dusp2^−/−^* HFD. Data are mean ± SD. White squares are *wt* mice fed SCD; Black squares are *dusp2^−/−^* mice fed SCD; White circles are *wt* mice fed HFD; Black circles are *dusp2^−/−^* mice fed HFD. Ns are 9, 8, 7 and 7 at 4 weeks and 9, 7, 6 and 7 at 12 weeks for *wt* SCD, *dusp2^−/−^* SCD, *wt* HFD and *dusp2^−/−^* HFD, respectively.

To assess glucose tolerance and insulin sensitivity, we performed oral glucose tolerance tests (OGTT) and assessed plasma insulin levels during the OGTT in both male and female mice after 4 and 12 weeks of either SCD or HFD. Compared with those fed the SCD, male mice fed the HFD were glucose intolerant (Figure 3a and d) and had higher plasma insulin levels, indicative of insulin resistance, during the OGTT (Figure 3 b, c, e and f) after both 4 and 12 weeks of HFD. No differences were observed in oral glucose tolerance or plasma insulin levels during the OGTT between *wt* and *dusp2^−/−^* male mice on either the SCD or HFD (Figure 3a–f). In female mice, 12 weeks of a HFD induced glucose intolerance and elevated plasma insulin levels compared to female mice fed the SCD (Figure 4a–f). As with the male mice, no differences were observed with oral glucose tolerance between *wt* and *dusp2^−/−^* female mice on either the SCD or HFD (Figure 4a and d). However, after 12 weeks of HFD, female *dusp2^−/−^* mice had lower plasma insulin levels during the OGTT than *wt* mice (Figure 4e and f), a finding indicative of an improvement in insulin sensitivity.

**Figure 5:**
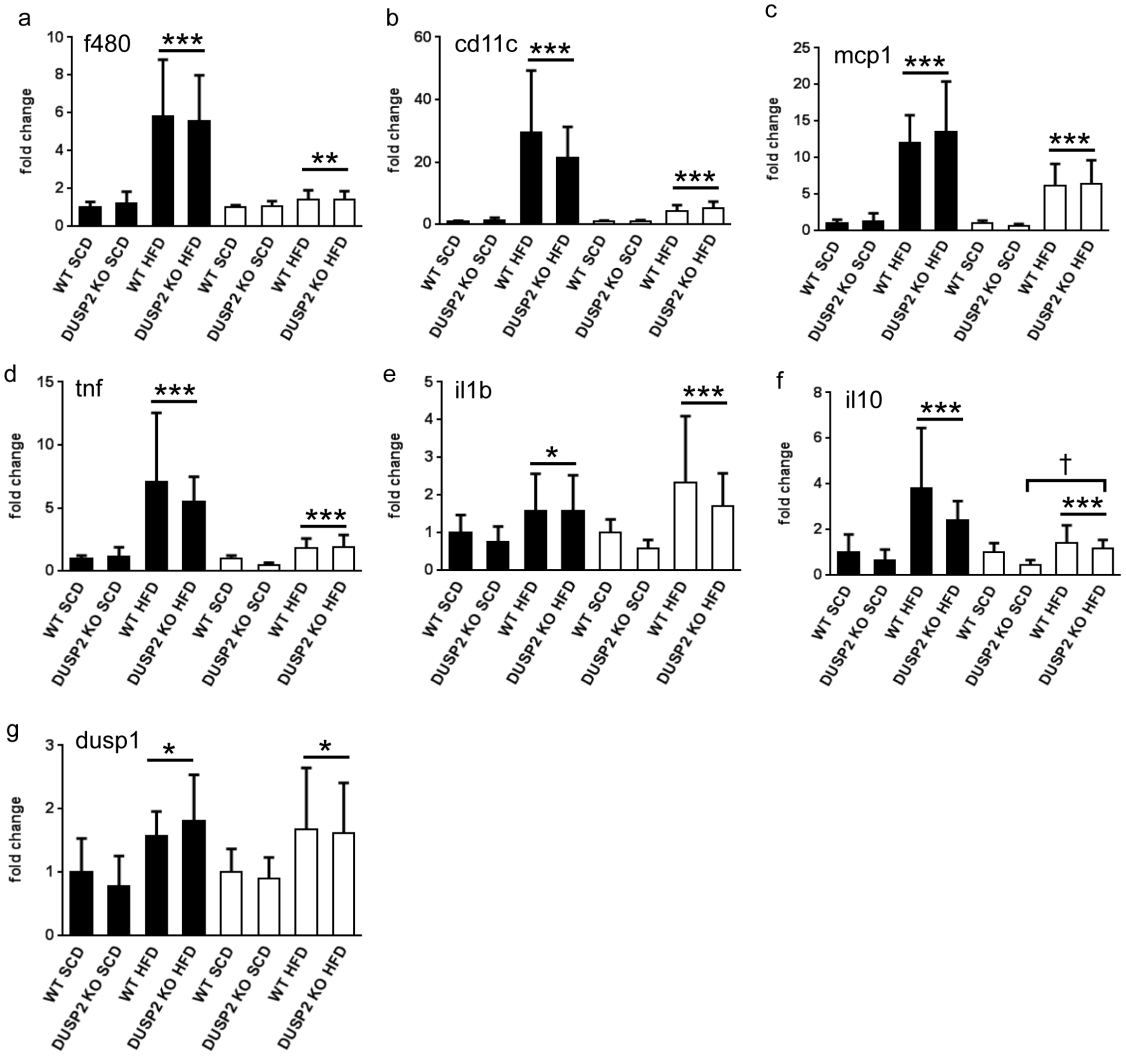
Inflammation in the WAT of *wt* and *dusp2^−/−^* male and female mice. The mRNA expression of (**a**) *f480*; (**b**) *cd11c*; (**c**) *mcp1*; (**d**) *tnf*; (**e**) *il1b*; (**f**) *il10* and (**g**) *dusp1* in epididymal adipose tissue. *p<0.05 Main Effect SCD vs. HFD; **p<0.01 Main Effect SCD vs. HFD; ***p<0.005 Main Effect SCD vs. HFD; †p<0.05 Main Effect *wt* vs. *dusp2^−/−^*. Data are mean ± SD. Black bars are male mice, white bars are female mice. Ns are 5, 8, 8 and 8 for male mice and Ns are 8, 8, 6 and 7 for female mice for *wt* SCD, *dusp2^−/−^* SCD, *wt* HFD and *dusp2^−/−^* HFD, respectively.

The data described thus far provided no evidence that an absence of DUSP2 activity in male mice affected obesity or measures of glucose tolerance and insulin sensitivity, whereas female *dusp2^−/−^* fed a HFD displayed a small, but significant improvement in insulin sensitivity when compared to *wt* controls. Given the published data ascribing an important role to DUSP2 in promoting inflammation and immune cell activation, we next investigated whether the absence of DUSP2 influenced epididymal adipose tissue inflammation. Total macrophage numbers, which included the recruitment of pro-inflammatory macrophages, inferred from the levels of *f480* and *cd11c* gene expression, were markedly elevated following 12 weeks of HFD (Figure 5a and b). Consistent with this finding, levels of gene expression for monocyte chemo-attractant protein 1 (*mcp1*), the pro-inflammatory cytokines tumour necrosis factor (*tnf*) and interleukin 1β (*il1b*), plus the anti-inflammatory cytokine *il10*, were all significantly higher following 12 weeks of HFD (Figure 5c–f), emphasizing the potent pro-inflammatory environment in adipose tissue induced by obesity. Although the HFD led to significant increases in the expression of *f480*, *cd11c*, *tnf*, *il1b* and *mcp*1 in female mice (Figures 5a–e), these differences were markedly lower than those observed in male mice. Notably, an absence of DUSP2 did not affect macrophage markers, pro-inflammatory cytokine or *il10* expression in male mice (Figures 5a–f). A near identical expression pattern for these genes was observed in female mice, the exception being *il10*, for which a small, but significant genotype-dependent difference was observed (Figure 5f). Furthermore, with the exception of *il1b*, the recruitment of macrophages and the increases in pro-inflammatory gene expression were markedly lower in female compared with male mice. This trend is consistent with the known anti-inflammatory effects of estrogen [24] and the finding that a loss of estrogen receptor α expression in the myeloid cells of female mice exacerbates obesity-associated WAT inflammation [25]. Finally, while DUSP1 expression was increased in both male and female mice following the HFD we observed no compensatory increases in DUSP1 expression in epididymal WAT of either male or female *dusp2^−/−^* mice (Figure 5g).

**Figure 6:**
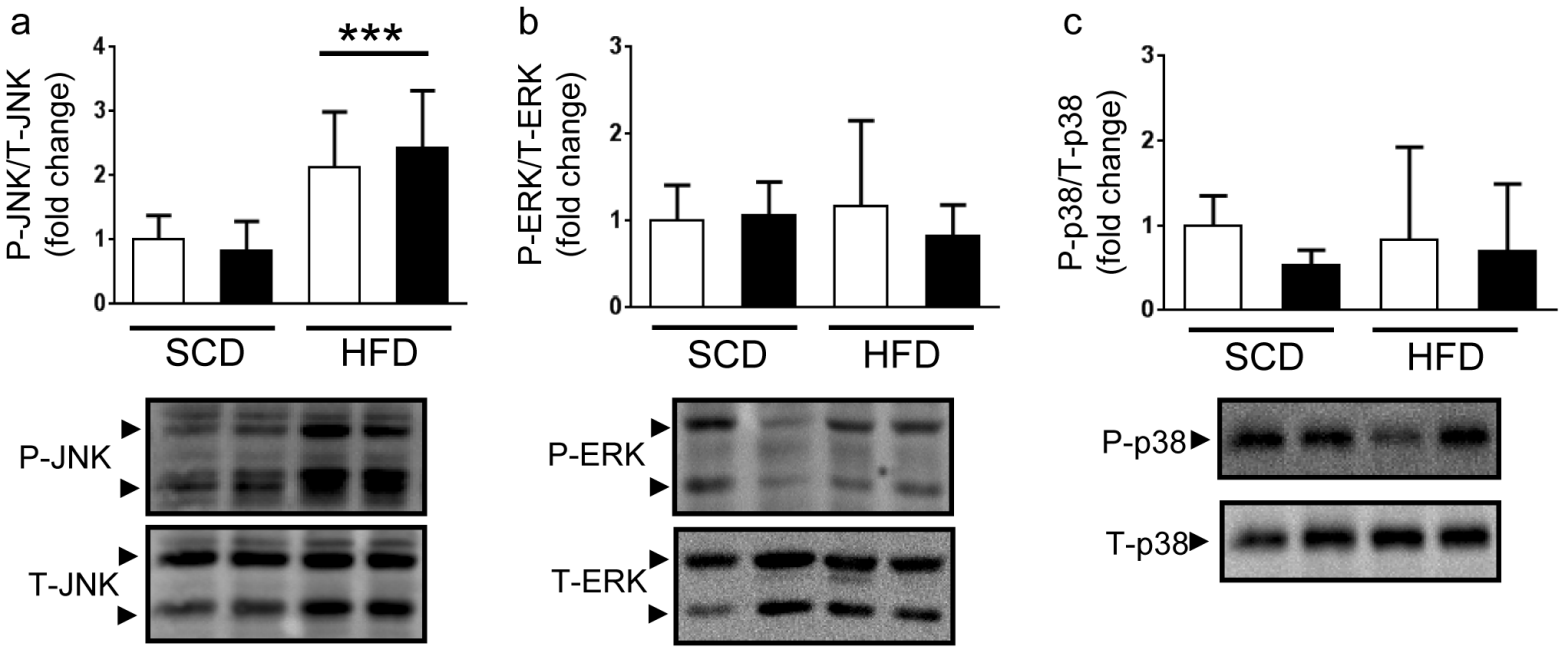
MAPK analysis in epididymal adipose tissue. JNK (**a**), ERK (**b**) and p38 (**c**) phosphorylation status in epididymal adipose tissue of male mice. ***p<0.005 Main Effect SCD vs. HFD. Data are mean ± SD. White bars are *wt* mice, black bars are *dusp2^−/−^* mice. Ns are 5, 7, 8 and 8 for *wt* SCD, *dusp2^−/−^* SCD, *wt* HFD and *dusp2^−/−^* HFD, respectively.

The activation of JNK and subsequent serine phosphorylation of insulin receptor substrate 1 (IRS1) in adipose tissue is postulated to be a key nexus by which a number of obesity-associated stimuli can directly impair insulin-dependent signaling [1]. A report that JNK phosphorylation is abnormally elevated in *dusp2^−/−^* cells following stimulation [18] prompted us to examine the impact of DUSP2 on JNK activation in epididymal adipose tissue. We observed a significant increase in the level of JNK phosphorylation in the WAT of male mice following 12 weeks of HFD (Figure 6a). However, the absence of DUSP2 had no additional impact on the levels of JNK phosphorylation in WAT (Figure 6a). We also determined ERK1/2 (Figure 6b) and p38 (Figure 6c) phosphorylation levels in WAT by western blotting. Neither the HFD nor DUSP2 deletion affected ERK1/2 or p38 phosphorylation in WAT.

**Figure 7:**
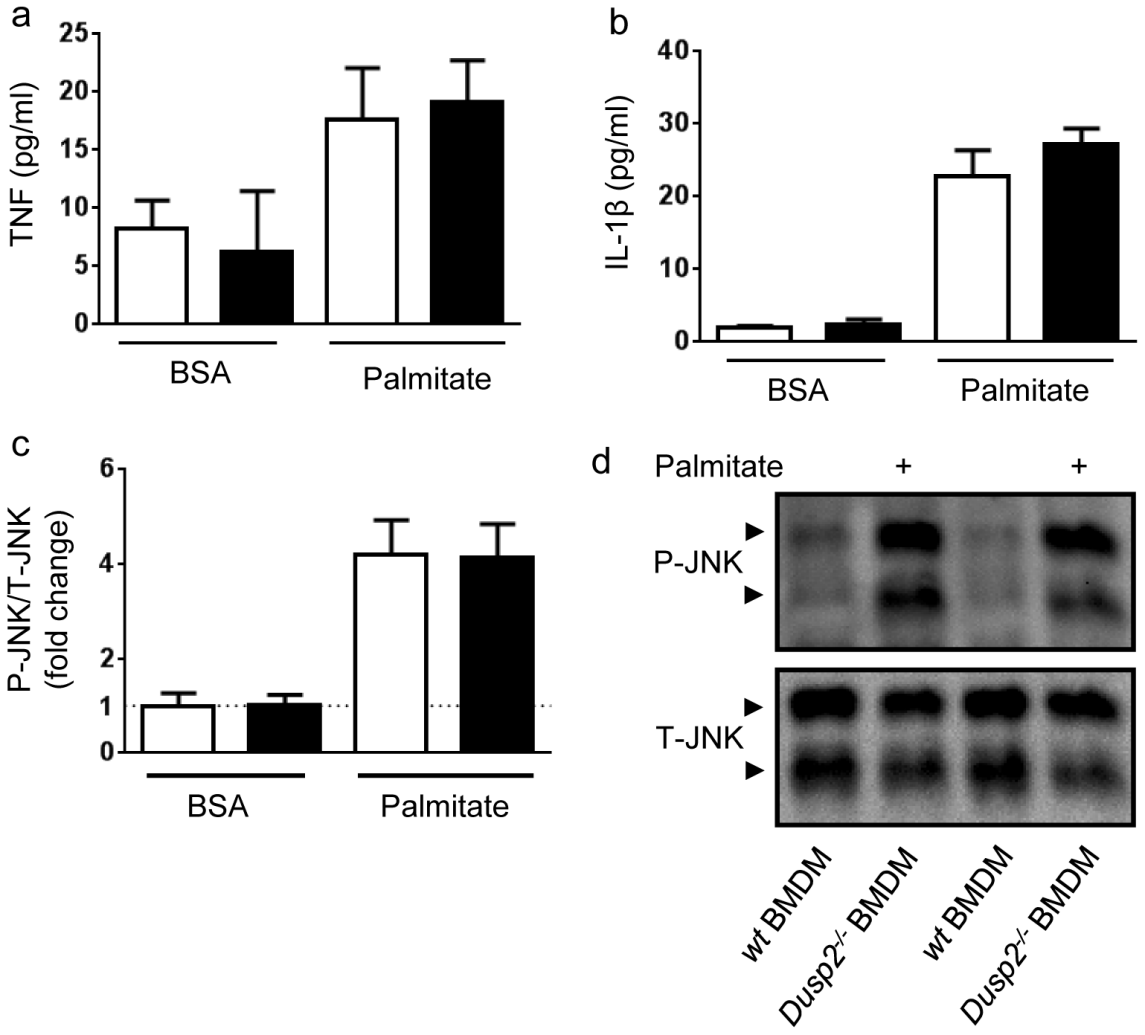
Treatment of BMDM from *wt* and *dusp2^−/−^* mice with the free fatty acid palmitate. BMDM were treated for 8 h (**a** and **b**) or 4 h (**c** and **d**) with either a control stimulus (BSA; white bars) or 1 mM Palmitate (black bars). **d** shows representative western blots from the quantifications shown in **c**. Data represent 3 separate mice per genotype each performed with 3 technical replicates. Data are mean ± SD.

A loss of DUSP2 activity in immune cells was previously reported to reduce LPS-induced pro-inflammatory cytokine production, but potentiate LPS-induced JNK phosphorylation [18]. Accordingly, we investigated whether pro-inflammatory cytokine production and JNK activation in BMDM activated by the long chain saturated fatty acid palmitate was also affected by an absence of DUSP2. While palmitate treatment resulted in increased TNF and IL-1β secretion as well as elevated JNK phosphorylation, we observed no differences in these responses between *wt* and *dusp2^−/−^* BMDM (Figure 7a–d).

## Discussion

Studies reporting roles for the MAPK dual specificity phosphatases DUSP1 and DUSP9 during inflammation, obesity and insulin sensitivity, prompted us to investigate what function DUSP2 might play in these processes. DUSP2, a nuclear specific DUSP that is mainly expressed in immune cells, had been shown in macrophages and mast cells to promote the production of various inflammatory mediators [18] that included key mediators of obesity-induced metabolic disease. Based on these properties of DUSP2, we examined whether obesity driven inflammation in WAT and its associated impact on glucose intolerance and insulin resistance would be reduced in mice lacking DUSP2. In contrast to the previously ascribed role for DUSP2 regulating inflammatory responses in culture and *in vivo*, our findings reported here demonstrate that DUSP2 plays no role in the development of obesity or obesity-associated inflammation. Furthermore, a lack of DUSP2 activity does not prevent the development of obesity-associated glucose intolerance or insulin resistance in male mice, although it does appear to have a small protective effect in female mice.

Previous studies have demonstrated roles for DUSP family members in the development of obesity, and obesity-associated inflammation and insulin sensitivity. DUSP1-deficient (*dusp1^−/−^*) mice, which exhibit elevated MAPK activity in several metabolically important tissues including skeletal muscle, liver and adipose tissue [20] have significantly less fat mass than control animals when fed a HFD for 12 weeks [20]. Nevertheless, they are still not protected from the deleterious effects a HFD has on glucose metabolism [20]. DUSP9 over-expression in genetically obese *ob*/*ob* mice on the other hand, improved glucose tolerance [19], while DUSP9 over-expression in 3T3-L1 adipocytes suppressed ERK1/2 and JNK activation, with a concomitant reduction in IRS1 serine phosphorylation that prevented the induction of insulin resistance [19]. A comparative evaluation of the biochemical properties of DUSP1 and DUSP9 with those of DUSP2, in conjunction with the impact their loss of function or over-expression has on obesity, inflammation and insulin sensitivity, provides valuable insight into the different mechanisms by which certain DUSPs, but not others, appear to regulate metabolism. For example, both DUSP1 and DUSP2, which are localized to the nucleus, are induced by stress or mitogenic signals and share overlapping substrate specificities for ERK and p38 [26], have different impacts on whole body metabolism, with DUSP1, but not DUSP2, controlling fat mass. This difference in the regulation of fat mass may reflect the broader pattern of DUSP1 expression and the ability of DUSP1 to also dephosphorylate JNK [27]. Although DUSP2 and DUSP9 can both dephosphorylate ERK, these DUSPs are restricted to the nucleus and cytoplasm respectively [27]. In the context of inflammation-induced insulin resistance seen in obesity, the subcellular localization of these DUSPs may be a critical point of difference, with serine phosphorylation of IRS1 by various serine kinases including ERK1/2 and JNK thought to be a key effector mechanism by which pro-inflammatory stimuli induce cellular insulin resistance [1]. As the phosphorylation of IRS1 occurs at the plasma membrane, it is DUSP9 rather than DUSP2 that has access to cytoplasmic ERK.

Given the importance of inflammation and the accumulation of numerous immune cell types in the WAT of obese mice, we hypothesized that a loss of DUSP2 function might reduce obesity-associated macrophage recruitment or inflammation in WAT. However, unlike the previous role ascribed to DUSP2 in positively regulating inflammatory responses, our data reveal that DUSP2 has no effect on obesity-associated inflammation. Several explanations could account for these differences in the roles of DUSP2 *in vivo*. For example, the qualitative and/or quantitative features of an inflammatory response may be important in dictating the involvement of DUSP2. Whereas DUSP2 was found to promote pathology in an arthritis model that rapidly induces leukocyte recruitment and pronounced inflammation within several days [18], obesity-associated inflammation in mice, by contrast, often takes weeks to months to manifest and is typically a low grade inflammatory response. Therefore, it is possible that the low-grade inflammation associated with obesity is not a sufficiently strong stimulus to employ DUSP2 as a means of modulating MAPK dependent inflammatory responses. Indeed, this may be reflected in the relatively modest increase in DUSP2 expression in the WAT of mice fed a HFD. Functional redundancy amongst the nuclear specific DUSPs, which in addition to DUSP2 include DUSP1, DUSP4 and DUSP5, may also be a contributing factor in DUSP2 not playing a unique role in obesity-associated WAT inflammation. Not only does DUSP2 share overlapping substrate specificity for MAPKs with certain of these other nuclear DUSPs [26], [27], it also has a similar pattern of expression with other nuclear DUSPs in activated leukocytes, in particular DUSP4 and DUSP5 [18]. Support for functional redundancy amongst the nuclear DUSPs comes from the recent report showing that in contrast to mice lacking either DUSP1 or DUSP4, which exhibit no cardiac pathology, mice missing both of these DUSPs develop cardiomyopathy due to unrestrained p38 activation [28].

While the absence of DUSP2 had no impact on obesity-associated inflammation and insulin resistance in male mice, we did observe a small, but significant improvement in the insulin sensitivity of female DUSP2-deficient mice fed the HFD. Of note, this improvement occurred irrespective of the absence of DUSP2 having no impact on HFD-induced obesity, immune cell recruitment or inflammation in the WAT. The increased insulin sensitivity in HF fed female *dusp2^−/−^* mice was modest, with these mice remaining markedly glucose intolerant and insulin resistant compared to mice fed the SCD. Irrespective of diet, we also observed that IL-10 gene expression was significantly lower in *dusp2^−/−^* female mice when compared to their *wt* counterparts. These changes while reproducible, were small, and in the absence of any other DUSP2-associated effect on other parameters of inflammation, suggests that this difference in IL-10 expression is unlikely to be of physiological significance.

With various PTPs, such as PTP1B [29], currently the subject of intense drug discovery efforts in the context of obesity and insulin resistance, specific DUSP family members found to control MAPK dependent obesity, inflammation and insulin resistance may represent potential targets in the treatment of metabolic disease. Using mice that lack DUSP2, a nuclear phosphatase shown to promote the production of pro-inflammatory cytokines, nitric oxide and prostaglandin E_2_ by activated macrophages and mast cells [18], we assessed whether DUSP2 represented a therapeutic target by which MAPK-dependent inflammatory gene expression associated with obesity could be suppressed. Our data provide compelling evidence that DUSP2 does not play a non-redundant role in the development of obesity or obesity-associated inflammation and its related pathologies, despite these conditions being underpinned by a strong inflammatory component.
